# Prognostic impact of presentation phenotypes in early‐stage favorable Hodgkin lymphoma

**DOI:** 10.1002/cncr.70532

**Published:** 2026-07-16

**Authors:** Hishan Tharmaseelan, Janina Jablonski, Ina Bühnen, Michael Fuchs, Johannes Rosenbrock, Hans T. Eich, Justin Ferdinandus, Christian P. Jaworek, Paul J. Bröckelmann, Peter Borchmann, Dennis A. Eichenauer

**Affiliations:** ^1^ Department I of Internal Medicine Center for Integrated Oncology Aachen Bonn Cologne Düsseldorf (CIO ABCD) Faculty of Medicine and University Hospital of Cologne University of Cologne Cologne Germany; ^2^ German Hodgkin Study Group (GHSG) Cologne Germany; ^3^ Department of Radiation Oncology and Cyberknife Center Faculty of Medicine and University Hospital Cologne University of Cologne Cologne Germany; ^4^ Department of Radiation Oncology University Hospital Münster Münster Germany

**Keywords:** Hodgkin lymphoma, lymph nodes, lymphoma, neoplasm staging, phenotype

## Abstract

**Background:**

In early‐stage favorable Hodgkin lymphoma (ES‐HL), lymph node involvement patterns (NIP) have not been investigated until now. Adding finer granularity to the current disease classification system evaluated through systematically incorporating NIP alongside established clinical variables may help to identify distinct patient subgroups and improve the understanding of ES‐HL.

**Methods:**

A total of 3715 patients with ES‐HL treated within the German Hodgkin Study Group HD10, HD13, and HD16 studies was retrospectively analyzed (stage I/II without risk factors). Involved lymph node regions were categorized into predefined anatomical areas, and the distribution patterns of affected lymph node regions were explored. Associations of patterns with baseline characteristics, histology, and 5‐year progression‐free survival (PFS) were analyzed using univariate and multivariable models.

**Results:**

Despite 22 possible combinations, 91.6% of patients presented with one of the six most common NIP. They differed in terms of age, sex distribution, weight, hemoglobin levels, and histologic subtype composition. For example, the combination of cervical and mediastinal involvement was more frequent in younger patients, females, enriched for nodular sclerosis histology, and associated with superior 5‐year PFS compared to other patterns (95.1% vs 91.9%, *p* < .001). Conversely, the combination of cervical and axillary involvement was associated with decreased 5‐year PFS (85.3% vs 93.4%, *p* < .001) and was enriched for male sex and nodular lymphocyte‐predominant Hodgkin lymphoma.

**Conclusions:**

Different presentation phenotypes exhibit distinct clinicopathologic characteristics and have prognostic impact in ES‐HL. Further studies integrating molecular profiling are warranted to elucidate root mechanisms and possibly refine risk group allocation.

## Graphical abstract

Using 3715 patients from the German Hodgkin Study Group HD10, HD13, and HD16 trials, we identified the most common lymph node involvement patterns in early‐stage favorable Hodgkin lymphoma. These presentation phenotypes differed markedly in terms of patient age, sex distribution, and histology. Distinct involvement patterns were associated with different baseline characteristics and progression‐free survival, indicating that anatomical presentation phenotype carries prognostic relevance in this otherwise favorable disease.

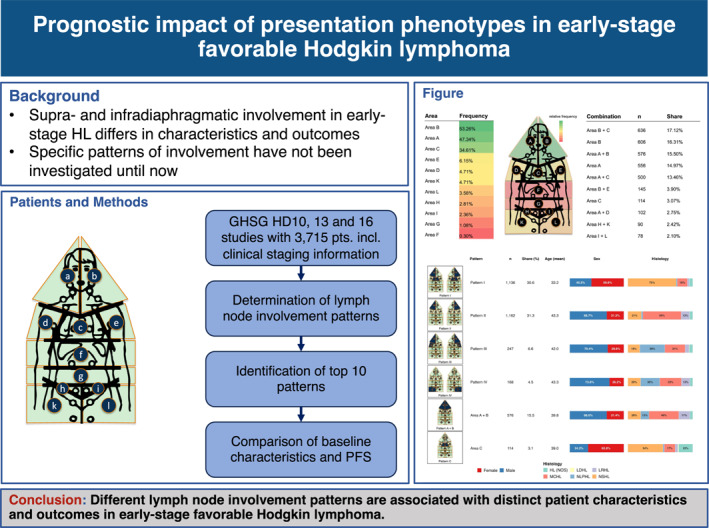

## INTRODUCTION

Most patients with early‐stage favorable Hodgkin lymphoma (ES‐HL), defined as stage I/II without risk factors, achieve long‐term remission when treated with a brief chemotherapy followed by consolidation radiotherapy (RT).

Based on the results from the randomized German Hodgkin Study Group (GHSG) HD10 study, two cycles of ABVD (doxorubicin, bleomycin, vinblastine, dacarbazine) followed by 20 Gy limited‐field RT represent the standard of care (SOC) for this patient group at most institutions.^1^ The 10‐year progression‐free survival (PFS) and overall survival (OS) rates with this approach were 87.2% and 94.1%, respectively.^2^ Although the majority of patients are likely cured, treatment failure occurs in approximately 15% of patients with ES‐HL. This indicates an unmet need for a better understanding of disease heterogeneity and risk stratification to inform modern therapeutic approaches.^3^

In patients diagnosed with ES‐HL, disease localization appears to be associated with certain clinical characteristics and impact outcomes. According to a previous analysis comprising patients treated within the GHSG HD13 and HD14 studies for ES‐HL and early‐stage unfavorable HL, respectively, infradiaphragmatic disease was associated with mixed cellularity (35.4%) and worse outcomes (infradiaphragmatic: 5‐year PFS 80.1% and OS 91.5% vs. supradiaphragmatic: 5‐year PFS 91.2% and OS 97.6%).^4^ Differences in terms of presentation characteristics were also described for patients with supradiaphragmatic and infradiaphragmatic diffuse large B‐cell lymphoma. As in HL, infradiaphragmatic disease was associated with an impaired prognosis also in diffuse large B‐cell lymphoma.^5^

In the era of precision medicine, the goal in HL research is to establish more personalized approaches beyond treatment guided by positron emission tomography and computed tomography or the use of targeted agents. To achieve this, an improved understanding of the disease, including the relationship between lymph node involvement patterns (NIP), histology, patient characteristics, and prognosis, is required.

We therefore conducted an analysis that aimed at identifying subgroups of ES‐HL with specific NIP, and to assess associated differences regarding patient characteristics, predominant histologic subtypes, and prognosis between these subgroups.

## METHODS

### Study design and participants

Patients aged 16 to 75 years enrolled in the randomized GHSG HD10, HD13, and HD16 studies [HD10: NCT00265018, HD13: ISRCTN63474366, and HD16: NCT00736320] for ES‐HL were included in the present analysis. ES‐HL was defined as stage I/II disease without clinical risk factors. Patients with stage IA nodular lymphocyte‐predominant HL (NLPHL) were excluded from participation in the studies. Details on chemotherapy, RT fields, and doses have been described elsewhere.^6^, ^7^, ^8^ The studies were conducted in accordance with the Declaration of Helsinki, and written informed consent was obtained from all patients.

### Definition of cohort and calculation of patterns

Disease localizations were determined based on lymph node regions that were recorded as affected in the baseline case report form. The following regions that constituted predefined lymph node areas were determined: area A/B (right/left cervical, periclavicular, and neck), area C (hilar and mediastinal), area D/E (right/left axillary), area F (upper abdominal, including celiac, splenic hilum, and liver hilum), area G (lower abdominal, including paraaortic and mesenteric), area H/I (right/left iliac), and area K/L (right/left inguinal and femoral) (Table 1). To reduce complexity, mirrored constellations (e.g., right‐only vs. left‐only axillary) were merged. This resulted in 22 distinct possible constellations. A detailed description of the contribution of lymph node regions to different lymph node areas is provided in Supplemental Material S1 and the derivation of the potential number of 22 possible patterns in Supplemental Material S2. NIP were defined according to the affected lymph node areas and their combinations when multiple areas were involved. Patients with missing staging information regarding the location patterns were excluded from the analysis.

**TABLE 1:** Lymph node areas.

A	Right	Cervical, periclavicular, and neck
B	Left	
C	Mediastinal and hilar	
D	Right	Axillary
E	Left	
F	Upper abdominal (including celiac, splenic hilum, and liver hilum)	
G	Lower abdominal (including paraaortic and mesenteric)	
H	Right	Iliac
I	Left	
K	Right	Inguinal and femoral
L	Left	

### Outcomes

To investigate associations between NIP and baseline characteristics, parameters including sex, age, histologic subtype (nodular sclerosis classic HL [NSHL], mixed cellularity classic HL [MCHL], lymphocyte‐depleted classic HL, lymphocyte‐rich classic HL, and NLPHL), body weight (kg), hemoglobin level (g/dL), and Ann Arbor stage were recorded and compared. In addition, possible associations between NIP and outcomes were evaluated by calculating and comparing the 5‐year PFS rates. PFS was defined as the time from enrollment until disease progression, relapse, or death from any cause and was censored at last follow‐up.

### Statistical analysis

Analyses were performed descriptively as no formal confirmatory testing was conducted in this explorative study. Nominal *p* values are provided to help interpret results. R (version 4.4.2) in RStudio (version 2024.12.0 + 467) was used to perform the computations. Patient characteristics for each NIP were summarized using the “tableone” package (version 0.13.2). To discover potential associations between patient demographics or clinical characteristics and the NIP, each pattern was compared to all other NIP using the chi‐squared test for categorical variables and Welch’s *t*‐test for continuous variables to account for unequal variances. PFS was analyzed using the Kaplan‐Meier method, comparing each pattern with all other patterns using the log‐rank test. Hazard ratios (HR), including 95% CI, are reported. To demonstrate the impact of NIP in the present era in a subgroup analysis, the 5‐year PFS rates were analyzed for a subset of patients who were treated with noninferior treatment approaches in comparison with the SOC (two cycles of ABVD followed by 20 Gy RT was considered as SOC; noninferior approaches included two cycles of ABVD + 30 Gy RT, and four cycles of ABVD + 20 or 30 Gy RT), whereas patients receiving treatment later shown to be inferior to the SOC (two cycles of ABV + 30 Gy IF, two cycles of ABVD + [positron emission tomography‐2‐guided RT], two cycles of AV[D] + 30 Gy IF) were excluded. For any observed effects, a Cox proportional hazards analysis was performed, comparing the influence of age, sex, and the specific pattern on PFS as compared to all other NIP. Kaplan‐Meier survival analysis and Cox proportional hazards regression modeling were performed using the “survival” package (version 3.8.3) and visualized with “survminer” (version 0.5.0). The proportional hazards (PH) assumption was tested using the Schoenfeld residuals test and confirmed by visual inspection. In an additional sensitivity analysis for patterns with a higher proportion of NLPHL cases (defined as >20%), and significant difference in terms of PFS, the analysis of SOC and SOC‐equivalent patients was repeated after exclusion of NLPHL to assess the impact of NLPHL histology on PFS.

## RESULTS

**FIGURE 1:**
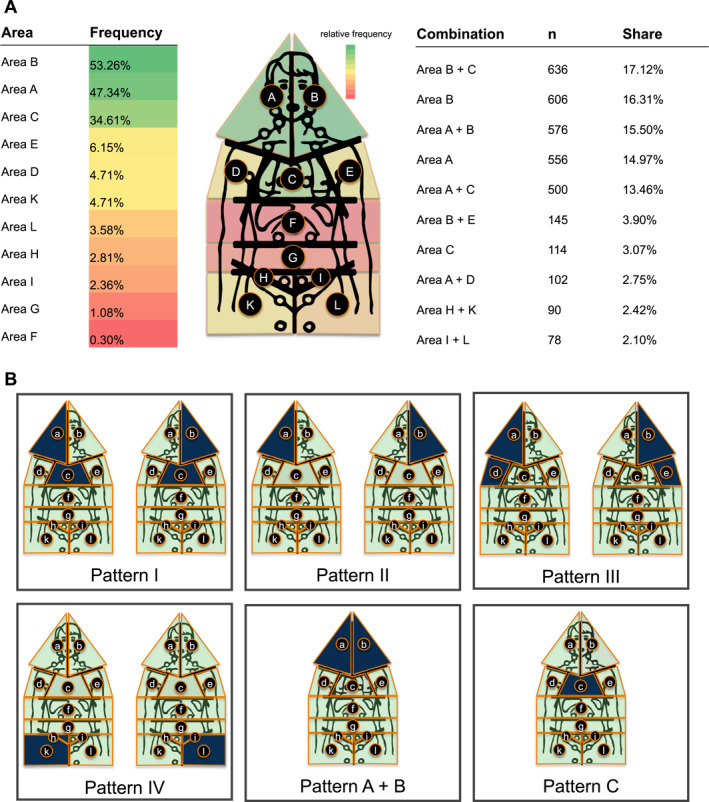
(A) Visualization of the frequency of disease areas and patterns. On the left, the frequency of each area in the total cohort is visualized, and the occurrence of patterns in all 3715 patients with ES‐HL. There can be multiple areas involved in a single patient. The visualization on the right side displays a heatmap of involved areas. (B) An overview of the six most common grouped disease patterns is displayed. ES‐HL indicates early‐stage Hodgkin lymphoma.

A total of 3715 patients were eligible for the analysis (Table 2). Of the 3831 patients available, 116 had been excluded (a CONSORT flow diagram is provided in the Supplemental Material S3). The mean age was 39.8 years (SD = 14.4), 40.9% (*n* = 1518) of patients were female, and 67.9% were classified as Ann Arbor stage II (*n* = 2521). Supradiaphragmatic lymph node areas were most frequently affected, with areas A and B (right/left cervical, periclavicular, and neck: 47.3% and 53.3%, respectively) being involved in almost half of all cases. Involvement of area C (hilar and mediastinal) was documented in 34.6% (*n* = 1286) of patients (Figure 1).

**TABLE 2:** Patient characteristics, split by trial.

		Overall	HD10	HD13	HD16
*N*		3715	1159	1455	1101
CS (*n*, %)	Unknown	2 (0.1)	2 (0.2)	0 (0.0)	0 (0.0)
	I	1192 (32.1)	372 (32.1)	475 (32.6)	345 (31.3)
	II	2521 (67.9)	785 (67.7)	980 (67.4)	756 (68.7)
Age, mean (SD)		39.78 (14.39)	38.71 (14.33)	40.78 (14.37)	39.57 (14.40)
Therapy (*n*, %)	2× ABV + 30 Gy IF	191 (5.1)	0 (0.0)	191 (13.1)	0 (0.0)
	2× ABVD + (20 Gy IF after pos. PET‐2)	547 (14.7)	0 (0.0)	0 (0.0)	547 (49.7)
	2× ABVD + 20 Gy IF	848 (22.8)	294 (25.4)	0 (0.0)	554 (50.3)
	2× ABVD + 30 Gy IF	835 (22.5)	286 (24.7)	549 (37.7)	0 (0.0)
	2× AV + 30 Gy IF	159 (4.3)	0 (0.0)	159 (10.9)	0 (0.0)
	2× AVD + 30 Gy IF	556 (15.0)	0 (0.0)	556 (38.2)	0 (0.0)
	4× ABVD + 20 Gy IF	289 (7.8)	289 (24.9)	0 (0.0)	0 (0.0)
	4× ABVD + 30 Gy IF	290 (7.8)	290 (25.0)	0 (0.0)	0 (0.0)
Sex (*n*, %)	Female	1518 (40.9)	454 (39.2)	592 (40.7)	472 (42.9)
	Male	2197 (59.1)	705 (60.8)	863 (59.3)	629 (57.1)

Abbreviations: A, Adriamycin (doxorubicin); B, bleomycin; CS, clinical stage; D, dacarbazine; HD10, HD13, HD16, German Hodgkin Study Group trials; IF, involved‐field radiotherapy; V, vinblastine.

**FIGURE 2:**
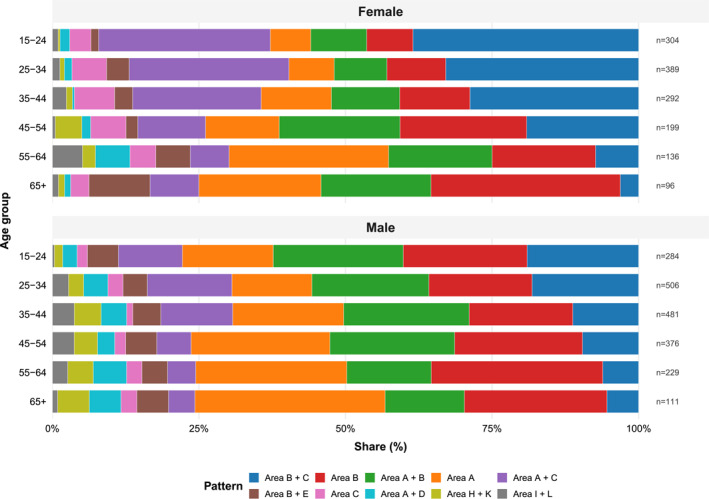
Distribution of the top 10 patterns between age groups, split by sex for (A) female and (B) male.

The most frequent NIP were areas B and C (17.1%, *n* = 636), followed by area B alone (16.3%, *n* = 606), areas A and B (15.5%, *n* = 576), area A alone (15.0%, *n* = 556), areas A and C (13.5%, *n* = 500), areas B and E (3.9%, *n* = 145), area C (3.1%, *n* = 114), areas A and D (2.7%, *n* = 102), areas H and K (2.4%, *n* = 90), and areas I and L (2.1%, *n* = 78) (Figure 2).

For analyses, mirrored patterns (i.e., left‐ and right‐sided constellations [e.g., H and K, I and L]) were grouped and analyzed as a single distribution profile, named patterns I–IV. Unique patterns were designated according to the involved areas (e.g., area C or A + B; Figure 1).

Pattern I was defined as an NIP of the unilateral cervical, periclavicular, or neck region, as well as hilar or mediastinal lymph nodes, affecting either area B and C or A and C (30.6%, *n* = 1136). Pattern II included unilateral involvement of cervical, periclavicular, or neck lymph nodes, affecting either area A or B (31.3%, *n* = 1162). Pattern III describes unilateral involvement of the right or left cervical, periclavicular, neck, and axillary lymph nodes, including either area A and D or B and E (6.6%, *n* = 247). Pattern IV is characterized by unilateral involvement of the right or left iliac and inguinal or femoral lymph nodes, including area I and L or H and K (4.5%, *n* = 168). Pattern C was defined by an isolated mediastinal involvement (area C) (3.1%, *n* = 114), whereas Pattern A + B described bilateral cervical, periclavicular, or neck involvement.

### Analysis of baseline features in the six most common patterns

**FIGURE 3:**
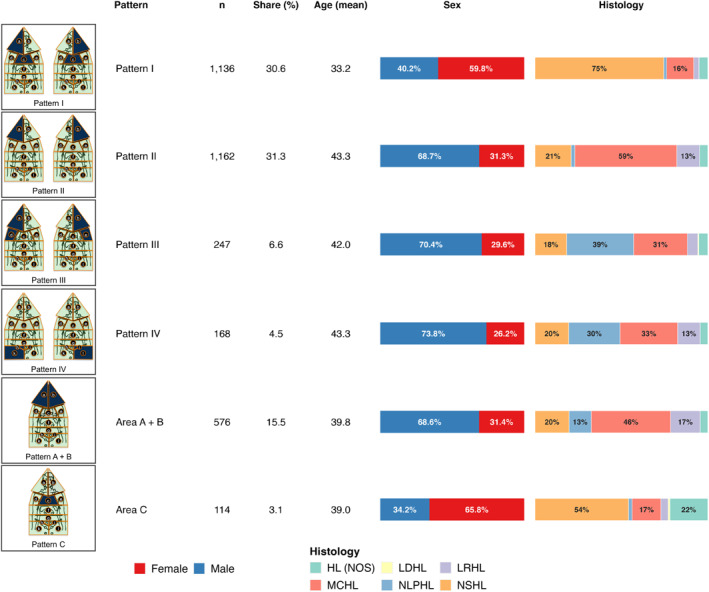
Overview of differing baseline characteristics in the top six patterns. HL indicates Hodgkin lymphoma; LDHL, lymphocyte‐depleted Hodgkin lymphoma; LRHL, lymphocyte‐rich Hodgkin lymphoma; MCHL, mixed cellularity Hodgkin lymphoma; NLPHL, nodular lymphocyte‐predominant Hodgkin lymphoma; NSHL, nodular sclerosis Hodgkin lymphoma; Share, share of total cohort *N* = 3715 (%).

An overview of the six most common NIP is displayed in Figure 3, and a detailed description is provided in the Supplemental Material S4. In this analysis, two female‐dominated NIP were identified: The proportion of women was higher in Pattern I (unilateral cervical, periclavicular, or neck, and hilar or mediastinal lymph nodes) compared to other patterns (59.8% [*n* = 679/1136] vs. 32.5% [*n* = 839/2579]), characterized also by a different histology (i.e., NSHL in 65.2% of cases [*n* = 741/1,136] vs. 19.0% of cases in other patterns [*n* = 490/2579], and observed in a younger patient population with a mean age of 33.2 years (SD = 11.6) (vs. 42.7 [SD = 14.6] in other patients). Hemoglobin levels and weight were lower in individuals presenting with Pattern I (Pattern I: Hb mean(M) = 13.7 g/dL [SD = 1.4]/M = 71.5 kg [SD = 16.0] vs. others: 14.4 g/dL [1.4]/80.6 kg [18.8]). The much less common Pattern C (isolated mediastinal involvement), which was only seen in 3.1% (*n* = 114) of cases with ES‐HL, was also associated with a higher proportion of women as compared to other NIP (65.8% [*n* = 75/114] vs. 40.1% [*n* = 1443/3601]), a different distribution of histologic subtypes (i.e., NSHL in 47.4% of Pattern C cases [*n* = 54/114] vs. 32.7% in other patterns [*n* = 1177/3601]) but no discernible difference compared to patients with other patterns in terms of age (M = 39.0 [SD = 13.8] vs 39.8 [14.4]). Similar to Pattern I, hemoglobin (M = 13.2 g/dL [SD = 1.5] vs. 14.2 g/dL [1.4]) and weight (M = 70.5 kg [SD = 18.0] vs. 78.0 kg [18.4]) were lower in Pattern C cases than in other cases.

Pattern II, characterized by unilateral involvement of cervical, periclavicular, or neck lymph nodes, presented with a higher proportion of male patients as compared to the other NIP (68.7% [*n* = 798/1162] vs. 54.8% [*n* = 1399/2553]) as well as an older age (M = 43.3 [SD = 15.0] vs. 38.2 [13.8]). Histologic subtype distribution differed between this pattern and all other NIP, with Pattern II being dominated by MCHL (52.3% [*n* = 608/1162] vs. 24.2% [*n* = 619/2553]). Patients with Pattern II were defined by higher weight (M = 79.9 kg [SD = 17.7] vs. 76.8 kg [18.7]) and hemoglobin levels (M = 14.5 g/dL [SD = 1.3] vs. 14.1 g/dL [1.4]) compared to patients with other NIP. Patients with Pattern A + B (bilateral cervical, periclavicular, or neck involvement) were, in contrast to patients with other NIP, distinguished by a higher proportion of males (68.6% [*n* = 395/576] vs. 57.4% [*n* = 1802/3139]) and different proportions of histologic subtypes with MCHL being most common (40.5% [*n* = 233/576] vs. 31.7% [*n* = 994/3,139]). Additionally, patients with this pattern had a higher weight (M = 80.9 kg [SD = 20.4] vs 77.2 kg 18) and a higher hemoglobin level (M = 14.6 g/dL [SD = 1.4] vs 14.1 g/dL [1.4]) compared to patients with other NIP, whereas their mean age did not differ (M = 39.8 [SD = 14.0] vs. 39.8 [14.5]).

Patients with Pattern III (unilateral involvement of the right or left cervical, periclavicular, neck, and axillary lymph nodes) tended to be older than patients with other NIP (M = 42.0 [SD = 15.0] vs. 39.6 [14.3]). In addition, the proportion of male patients was higher (70.4% [*n* = 174/247] vs. 58.3% [*n* = 2023/3468]), and the distribution of histologic subtypes differed in comparison with other NIP. Pattern III was enriched for patients with NLPHL (34.0% [*n* = 84/247] vs. 5.6% [*n* = 194/3468]). As in Pattern II (unilateral involvement of cervical, periclavicular, or neck lymph nodes) and Pattern A + B (bilateral cervical, periclavicular, or neck involvement), weight (M = 82.2 kg [SD = 17.6] vs 77.5 [18.5]) and hemoglobin level (M = 14.5 g/dL [SD = 1.2] vs 14.2 g/dL [1.4]) were higher in patients with Pattern III than in patients with other patterns.

In Pattern IV (unilateral involvement of the right or left iliac and inguinal or femoral lymph nodes), the only infradiaphragmatic pattern among the six major NIP, patients were older than in other patterns (M = 43.3 [SD = 12.8] vs. 39.6 [14.4]) and it was characterized by a higher proportion of males (73.8% [*n* = 124/168] vs. 58.4% [*n* = 2,073/3,547]). As in Pattern III (unilateral involvement of the right or left cervical, periclavicular, neck, and axillary lymph nodes), NLPHL was more frequent in this pattern than in other patterns (24.4% [*n* = 41/168] vs. 6.7% [*n* = 237/3547]). The same was true for higher weight (M = 87.8 kg [SD = 18.7] vs 77.3 kg [18.3]) and hemoglobin levels (M = 14.6 g/dL [SD = 1.3] vs 14.2 g/dL [1.4]).

The relationship between NIP and Ann Arbor stage deserves consideration. Single‐area patterns (Pattern II, Area C) are predominantly stage I (76.9% and 71.1%, respectively), whereas two‐area patterns almost exclusively occur in stage II patients (98.4%–100%; Supplemental Material S5).

### Impact of patterns on PFS

The subgroup analysis for the population receiving SOC or noninferior treatments included a total of 2262 patients. Patients with Pattern I had a better 5‐year PFS as compared to other NIP (Pattern I: 95.1%; 95% CI, 93.4–96.8 vs. other patterns: 91.9%; 95% CI, 90.4–93.4; *p* < .001). Age remained associated with an impaired PFS (HR = 1.04 per year; 95% CI, 1.03–1.05; *p* < .001), whereas male sex was not (HR = 1.19; 95% CI, 0.90–1.57; *p* = .23). The PH assumption was confirmed.

Patients with Pattern III had a worse PFS than patients with other NIP (Pattern III: 85.3%; 95% CI, 79.3–91.7 vs. other patterns: 93.4%; 95% CI, 92.3–94.6; *p* < .001). The Cox model indicated an increased hazard for Pattern III (HR = 2.07; 95% CI, 1.37–3.13; *p* < .001). Age was again associated with a worse PFS (HR = 1.04 per year; 95% CI, 1.03–1.05; *p* < .001), whereas male sex was not (HR = 1.17; 95% CI, 0.88–1.54; *p* = .27). The PH assumption was confirmed.

**FIGURE 4:**
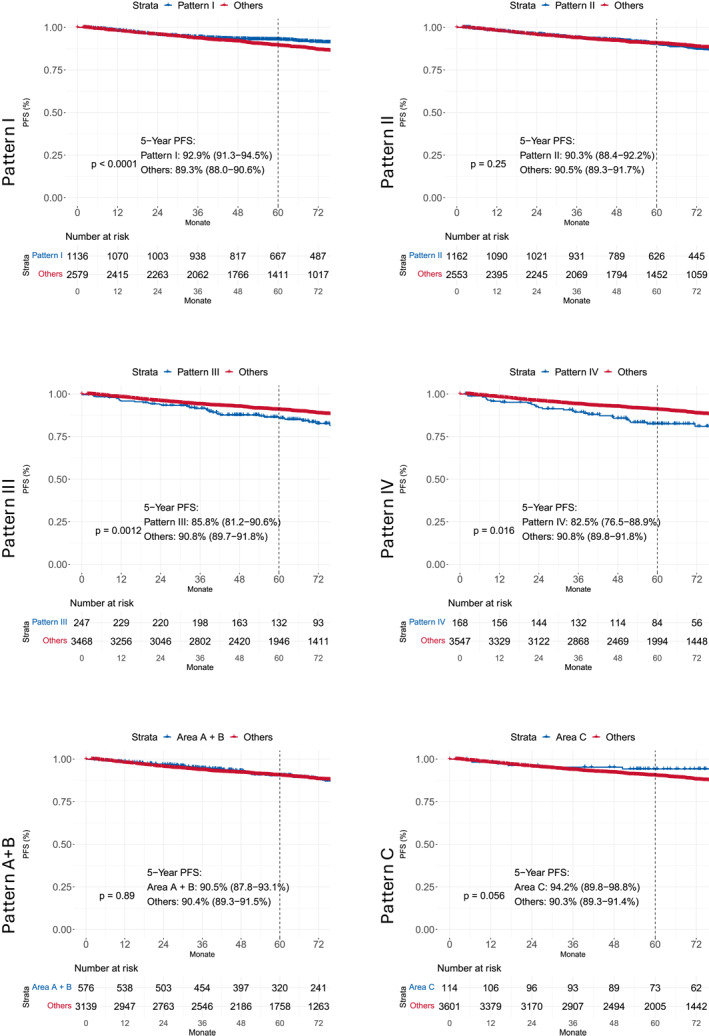
Progression‐free survival over all patients by patterns I‐IV, A + B and C in the SOC or noninferior treatment cohort (including 2× ABVD + 20 Gy IF, 2× ABVD + 30 Gy IF, 4× ABVD + 20 Gy IF, 4× ABVD + 30 Gy IF).

Pattern C was associated with a better 5‐year PFS compared to other NIP (96.9%; 95% CI, 92.8–100.0 vs. others: 92.8%; 95% CI, 91.6–93.9; *p* = .036). In the Cox regression, age was the only predictor associated with increased hazard for a PFS event (HR = 1.04 per year; 95% CI, 1.03–1.05; *p* < .001). For all other NIP, no difference in terms of PFS was observed (Figure 4).

### Sensitivity analysis excluding NLPHL

In a sensitivity analysis after exclusion of all patients with NLPHL from the SOC/noninferior subset (*n* = 2097), the association of Pattern III with inferior PFS persisted (5‐year PFS 85.6%; 95% CI, 78.6–93.3 vs others 93.7%[ 95% CI, 92.6–94.9; *p* < .001). In the Cox regression, the pattern remained predictive with an increased hazard (HR for Pattern III: 2.08; 95% CI, 1.29–3.35; *p* < .01) (Supplemental Material S6).

## DISCUSSION

In this study, distinct disease phenotypes in ES‐HL with differing clinicopathologic characteristics were identified through analysis of NIP. The patterns differed in terms of age, sex, weight, hemoglobin levels, and histologic subtype composition. Although there are 22 possible combinations, 91.6% of patients fit into one of six major patterns showing a strong concentration of conserved frequent disease presentation phenotypes. Only three patterns were associated with PFS outcomes that differed in comparison with all other NIP: Pattern I and Pattern C were associated with a superior and Pattern III with an impaired PFS.

The results of the present analysis emphasize that HL sometimes resembles primary mediastinal B‐cell lymphoma (PMBCL).^9^, ^10^ PMBCL is characterized by a female predominance (56.3%) and a similar age at diagnosis (median age, 32.8 years) as compared to HL.^11^ Molecular analyses of mediastinal HL and PMBCL support the hypothesis that comparable patterns of disease involvement possibly reflect biological similarities.^9^ In this analysis, Pattern C (isolated involvement of mediastinal/hilar nodes) showed a high proportion of female patients and was frequently associated with NSHL. The enrichment of NSHL and the younger, predominantly female patient population in Pattern I corresponds to epidemiologic features of supradiaphragmatic HL described previously. In this group, favorable PFS was observed, which is consistent with earlier data exhibiting excellent long‐term disease control in patients with upper mediastinal and cervical disease presentation.^4^

Pattern II and Pattern A + B mirror characteristics of HL with higher MCHL proportions, male predominance, and higher hemoglobin and weight. Both patterns had no prognostic impact. Pattern III represents the biologically most distinctive NIP as it is strongly associated with NLPHL. Additional characteristics such as older age and a pronounced male predominance reflect well‐described epidemiologic signatures of NLPHL.^12^

Pattern IV corroborates known features of infradiaphragmatic disease: older age, male predominance, and higher proportion of NLPHL cases.^4^ The superior PFS observed in female‐dominated patterns, and the inferior PFS in male‐dominated patterns, is consistent with male sex being an unfavorable prognostic factor in the International Prognostic Score.^13^

Our results show that the NIP partially partitions the cohort along the stage I/II boundary. However, the prognostic value of NIP consists in its capacity to stratify patients within the heterogeneous stage II group, which encompasses anatomically diverse presentations, from contiguous cervical–mediastinal involvement to noncontiguous infradiaphragmatic combinations, that, as demonstrated here, carry distinct clinicopathologic and prognostic profiles. NIP thus provides anatomical resolution beyond what the binary stage I/II distinction can offer.

Although NLPHL accounts for only ∼5% of all HL cases, it was markedly enriched in Pattern III (34%) and Pattern IV (24.4%). These distributional and outcome differences point to the distinct disease biology of NLPHL as compared to the histologic subtypes of cHL.^14^ Pattern III was associated with inferior PFS, and this prognostic association persisted after exclusion of all NLPHL cases, demonstrating that the prognostic signal in Pattern III is not solely driven by NLPHL biology.

A key unresolved question in HL is the biological basis underlying the two distinct age‐related incidence peaks observed between 15 and 30 years and older than 55 years.^15^ The age‐associated distribution of histologic subtypes suggests that HL may represent at least two distinct but overlapping disease entities. In addition to the reduced physiological fitness typically associated with aging, molecular differences may strongly influence disease presentation and treatment responses across age groups. These molecular variations could contribute to divergent clinical trajectories and outcomes, underscoring the need for further investigation of age‐related biological factors in lymphoid malignancies. Biological evidence supports this hypothesis. For example, latent membrane protein 1 expression has been linked to older age and an impaired prognosis,^16^ and Epstein–Barr virus DNA is detected more frequently in older patients with cHL.^17^, ^18^ It is known that MCHL is associated with Epstein–Barr virus regardless of age.^19^ MCHL‐dominated patterns are those involving cervical and periclavicular lymph nodes (Pattern II/A + B).

Strengths of the present analysis include the large, prospectively collected cohort from three randomized GHSG trials, as clinical trial data undergo quality assurance, pathology expert review, and homogeneous treatment, allowing robust pattern‐based comparisons. The systematic, anatomically grounded classification of lymph node areas provides the first high‐resolution approach to phenotype ES‐HL according to dissemination pathways.

Despite the mentioned strengths, there are also limitations. For instance, PFS comparisons are challenging, as the number of patients per group decreases significantly when stratified by patterns and restricted to those treated with SOC or noninferior approaches, respectively. Thus, further studies with standardized staging methods (incorporating positron emission tomography and computed tomography as a mandatory standard) and larger subgroup samples are needed to validate the findings. In addition, the high number of possible pattern combinations across multiple involved areas dilutes the statistical power of each subgroup analysis. Although the prognostic associations between NIP and PFS seem to be robust, additional translational work, in particular molecular profiling of the Pattern III subgroup, is required.

The findings on disease patterns in ES‐HL suggest similar analyses also in early‐stage unfavorable and advanced‐stage HL to determine whether associations between disease patterns and clinicopathologic features persist across risk groups. With the incorporation of novel agents (e.g. immune checkpoint inhibitors) in limited‐stage HL,^20^, ^21^ the outcome with such new approaches according to presentation patterns should be examined. In addition, the relevance of the distance between lesions, as demonstrated in positron emission tomography radiomics analyses in relapsed and refractory HL by Driessen et al.,^22^ can be included by the approximation using areas to develop a clinically feasible prognostication tool.

Taken together, the findings from the present analysis demonstrate that anatomical involvement patterns capture biologically different HL phenotypes with distinct prognostic features and outcomes. They may serve as a springboard for future classification systems beyond the current Ann Arbor–based system through integrating clinical, histologic, and possibly molecular characteristics.

## AUTHOR CONTRIBUTIONS

**Hishan Tharmaseelan**: Conceptualization; methodology; formal analysis; investigation; data curation; writing—original draft; visualization. **Janina Jablonski**: Investigation; data curation. **Ina Bühnen**: Investigation; data curation. **Michael Fuchs**: Formal analysis; software. **Johannes Rosenbrock**: Investigation; resources. **Hans T. Eich**: Resources; writing—review & editing. **Justin Ferdinandus**: Writing—review & editing; investigation. **Christian P. Jaworek**: Investigation; writing—review & editing. **Paul J. Brockelmann**: Conceptualization; writing—review & editing. **Peter Borchmann**: Conceptualization; supervision; writing—review & editing. **Dennis A. Eichenauer**: Conceptualization; methodology; supervision; project administration; writing—review & editing.

## CONFLICT OF INTEREST STATEMENT

Johannes Rosenbrock reports honoraria from Takeda and MSD. Paul J. Bröckelmann reports advisory roles or consultancy for Hexal, Merck Sharp & Dohme (MSD), Need Inc., Stemline, and Takeda; stock options in Need Inc.; honoraria from AstraZeneca, BeiGene, Bristol‐Myers, Squibb/Celgene (BMS), Eli Lilly, MSD, Need Inc., Roche, Stemline, and Takeda; and institutional research funding from BeiGene, BMS, MSD, and Takeda. All other authors report no conflicts of interest.

## PATIENT CONSENT STATEMENT

Written informed consent was obtained from all patients enrolled in the GHSG HD10, HD13, and HD16 studies.

## Supporting information

Supplementary Material

## Data Availability

The data that support the findings of this study are available on request from the corresponding author. The data are not publicly available due to privacy or ethical restrictions.
