# Supplementary material for: Prognostic impact of presentation phenotypes in early‐stage favorable Hodgkin lymphoma
Source: Cancer. 2026 Jul 16;132(14):e70532. doi: 10.1002/cncr.70532 (PMC13376989; doi:10.1002/cncr.70532)
Supplement: Supplementary file 1 — Supplementary Material [file CNCR-132-e70532-s001.docx]

**Supplement S1: Area to pattern**

| **Region_ID** | **Ann-Arbor-Region** | **Area** |
| --- | --- | --- |
| 1 | Waldeyer's lymphatic ring left | a |
| 2 | Waldeyer's lymphatic ring right | b |
| 3 | Upper cervical/ submandibular left | a |
| 4 | Upper cervical/ submandibular right | b |
| 5 | Cervical left | a |
| 6 | Cervical right | b |
| 7a | Supraclavicular right | b |
| 7b | Infraclavicular right | b |
| 8a | Supraclavicular left | a |
| 8b | Infraclavicular left | a |
| 9 | Axillary right | d |
| 10 | Axillary left | e |
| 11a | Upper mediastinum | c |
| 11b | Lower mediastinum | c |
| 12 | Pulmonary hilum right | c |
| 13 | Pulmonary hilum left | c |
| 14 | Lung left | Organ |
| 15 | Lung right | Organ |
| 16 | Liver | Organ |
| 17a | Liver hilum | f |
| 17b | Coeliac | f |
| 18 | Mesenterial | g |
| 19 | Spleen | f |
| 20 | Splenic hilum | f |
| 21 | Paraaortic | g |
| 22 | Iliac right | h |
| 23 | Iliac left | i |
| 24 | Inguinal/ femoral right | k |
| 25 | Inguinal/ femoral left | l |
| 26 | Bone | Organ |
| 27 | Bone Marrow | Organ |
| 28 | Pleura | Organ |
| 29 | Pericardium | Organ |
| 30 | Other nodal location | Organ |

**Supplement S2: Definition of nodal involvement patterns**

Patterns were classified according to involvement of predefined anatomical regions (A–L) and grouped into either supradiaphragmatic (A–E) or infradiaphragmatic (F–L) distributions, separated by the boundary between regions C and F. Only patterns involving one or two regions were considered. To avoid redundant counting, mirror-symmetric left/right constellations were treated as one.

| **Region** | **Pattern class** | **Mirror-equivalent patterns** |
| --- | --- | --- |
| Supra | A | B |
| Supra | D | E |
| Supra | C | - |
| Supra | A+B |  |
| Supra | D+E |  |
| Supra | A+C | B+C |
| Supra | A+D | B+E |
| Supra | A+E | B+D |
| Supra | C+D | C+E |
| Infra | F | - |
| Infra | G | - |
| Infra | H | I |
| Infra | K | L |
| Infra | F+G |  |
| Infra | F+H | F+I |
| Infra | F+K | F+L |
| Infra | G+H | G+I |
| Infra | G+K | G+L |
| Infra | H+I |  |
| Infra | H+K | I+L |
| Infra | H+L | I+K |
| Infra | K+L |  |

**Supplement S3: Consort flow diagram**

**
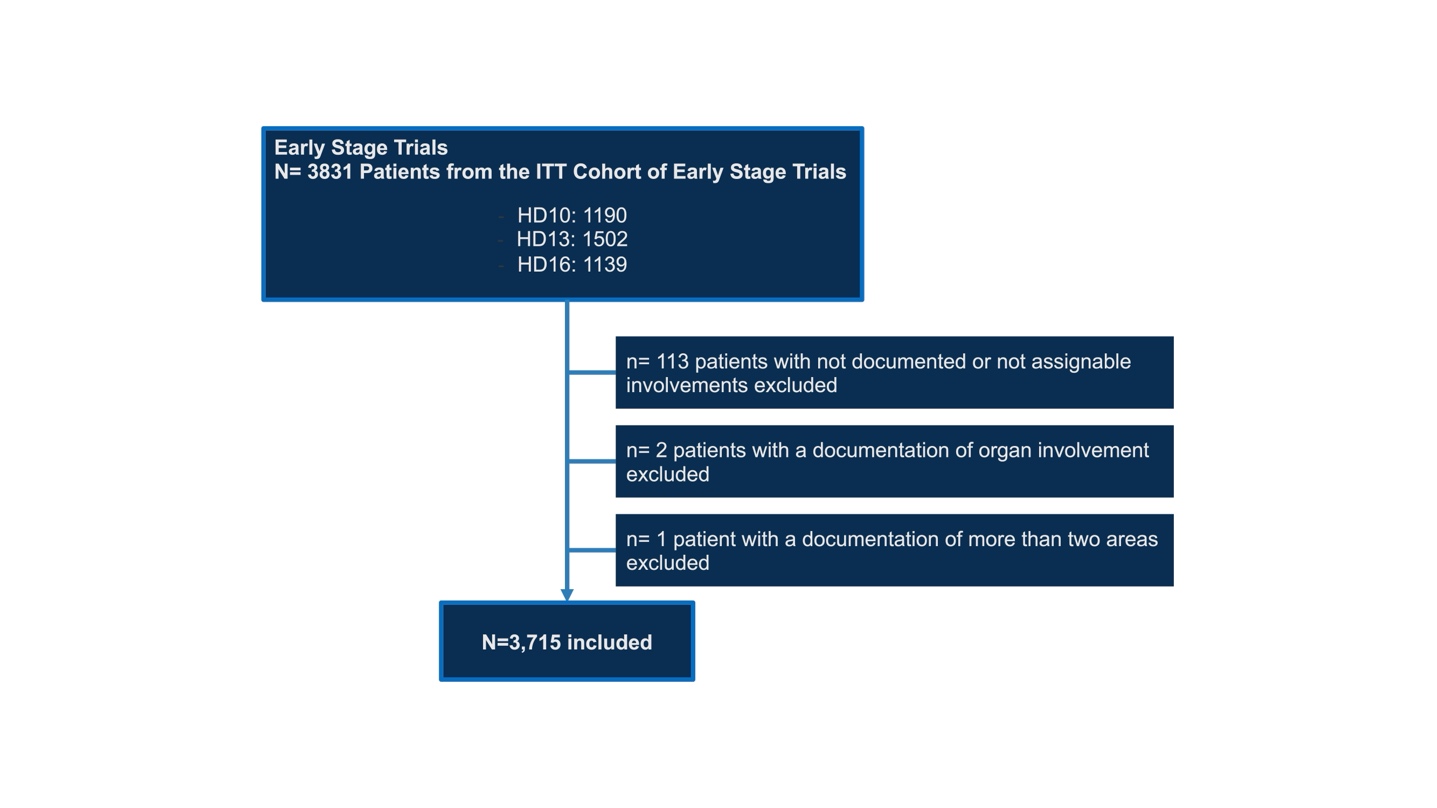
**

**Supplement S4: Overview of the baseline features in the top six patterns**

**Supplement S5: Stage I vs II distribution per Nodal Involvement Pattern**

| **GroupedPattern** | **n_I** | **n_II** | **pct_I** | **pct_II** |
| --- | --- | --- | --- | --- |
| **Area A + B** | 9 | 567 | 1.6 | 98.4 |
| **Area C** | 81 | 33 | 71.1 | 28.9 |
| **Pattern I** | 3 | 1133 | 0.3 | 99.7 |
| **Pattern II** | 893 | 269 | 76.9 | 23.1 |
| **Pattern III** | 0 | 247 | 0 | 100 |
| **Pattern IV** | 2 | 166 | 1.2 | 98.8 |

**Supplement S6: Sensitivity analysis - NLPHL excluded**

| **Pattern** | **n (total)** | **n (NLPHL)** | **Share (%, NLPHL)** |
| --- | --- | --- | --- |
| Area A + B | 576 | 65 | 11.28% |
| Area C | 114 | 2 | 1.75% |
| Pattern I | 1136 | 16 | 1.41% |
| Pattern II | 1162 | 23 | 1.98% |
| Pattern III | 247 | 84 | 34.01% |
| Pattern IV | 168 | 41 | 24.40% |
